# Supplementary material for: Imperfect Bayesian inference in visual perception
Source: PLoS Comput Biol. 2019 Apr 18;15(4):e1006465. doi: 10.1371/journal.pcbi.1006465 (PMC6472731; doi:10.1371/journal.pcbi.1006465)
Supplement: S1 Appendix — (DOCX) [file pcbi.1006465.s001.docx]

Computational imperfections in human visual search

Elina Stengård & Ronald van den Berg

S1 Appendix: Supplementary Methods

**Derivation of the optimal decision variable for the visual search task**

We denote target presence by a binary variable *T* (0=absent, 1=present), set size by *N*, the mean of the target distribution by *μ*_T_, the width of the stimulus distributions by *σ*_ext_, the stimulus values on a given trial by **s**={*s*_1_, *s*_2_, …, *s_N_*}, the location (index) of the target by *L*, and the observer’s noisy observations of the stimulus values by **x**={*x*_1_, *x*_2_, …, *x_N_*}. The Bayesian optimal observer reports “target present” if the posterior probability of target presence exceeds that of target absence, *p*(*T*=1|**x**) > *p*(*T*=0|**x**). This strategy is equivalent to reporting “target present” if the log posterior ratio exceeds 0,

$$d\left(\mathbf{x}\right)\equiv\log\frac{p\left(T=1|\mathbf{x}\right)}{p\left(T=0|\mathbf{x}\right)}>0.$$

where *d*(**x**) is referred to as the decision variable. Applying Bayes' rule, we find

$$d\left(\mathbf{x}\right)=\log\frac{p\left(\mathbf{x}|T=1\right)}{p\left(\mathbf{x}|T=0\right)},$$

where we made use of the fact that *p*(*T*=0)=*p*(*T*=1) in all our experiments. Using basic rules of probability, we rewrite the numerator to

$$p\left(\mathbf{x}|T=1\right)=\frac{p\left(\mathbf{x},T=1\right)}{p\left(T=1\right)}=\frac{\sum_{\hat{L}=1}^{N} \int\cdots\int p\left(\mathbf{s},\mathbf{x},T=1,L=\hat{L}\right)ds_{1}\cdots ds_{N}}{p\left(T=1\right)}.$$

Applying our knowledge of the generative model (S1 Fig), this evaluates to

$$\begin{matrix} =\sum_{\hat{L}=1}^{N} \int\cdots\int p\left(\mathbf{x}|\mathbf{s}\right)p\left(\mathbf{s}|L=\hat{L},T=1\right)p\left(L=\hat{L}\right)ds_{1}\cdots ds_{N} \\ =\frac{1}{N}\sum_{\hat{L}=1}^{N} \int\cdots\int G\left(x_{\hat{L}};s_{\hat{L}},\sigma_{\hat{L}}\right)G\left(s_{\hat{L}};\mu_{\text{T}},\sigma_{\text{ext}}\right)\prod_{i\neq\hat{L}} G\left(x_{i};s_{i},\sigma_{i}\right)G\left(s_{i};-\mu_{\text{T}},\sigma_{\text{ext}}\right)ds_{1}\cdots ds_{N} \\ =\frac{1}{N}\sum_{\hat{L}=1}^{N} \int G\left(x_{\hat{L}};s_{\hat{L}},\sigma_{\hat{L}}\right)G\left(s_{\hat{L}};\mu_{\text{T}},\sigma_{\text{ext}}\right)ds_{\hat{L}}\prod_{i\neq\hat{L}} \int G\left(x_{i};s_{i},\sigma_{i}\right)G\left(s_{i};-\mu_{\text{T}},\sigma_{\text{ext}}\right)ds_{i}, \end{matrix}$$

where *G*(*x*; *μ*, *σ*) is a Gaussian distribution with mean *μ* and standard deviation *σ*. The denominator of Eq. evaluates to

$$\begin{matrix} p\left(\mathbf{x}|T=0\right)=\frac{p\left(\mathbf{x},T=0\right)}{p\left(T=0\right)}=\frac{\int\cdots\int p\left(\mathbf{x},T=0,\mathbf{s}\right)ds_{1}\cdots ds_{N}}{p\left(T=0\right)} \\ =\int\cdots\int p\left(\mathbf{x}|\mathbf{s}\right)p\left(\mathbf{s}|T=0\right)ds_{1}\cdots ds_{N} \\ =\prod_{i=1}^{N} \int G\left(x_{i};s_{i},\sigma_{i}\right)G\left(s_{i};-\mu_{\text{T}},\sigma_{\text{ext}}\right)ds_{i}. \end{matrix}$$

Combining Eqs. , , and yields the decision variable as presented in the main text:

$$\begin{matrix} d\left(\mathbf{x}\right)=\log\frac{\frac{1}{N}\sum_{\hat{L}=1}^{N} \int G\left(x_{\hat{L}};s_{\hat{L}},\sigma_{\hat{L}}\right)G\left(s_{\hat{L}};\mu,\sigma_{\text{ext}}\right)ds_{\hat{L}}\prod_{i\neq\hat{L}} \int G\left(x_{i};s_{i},\sigma_{i}\right)G\left(s_{i};-\mu_{\text{T}},\sigma_{\text{ext}}\right)ds_{i}}{\prod_{i=1}^{N} \int G\left(x_{i};s_{i},\sigma_{i}\right)G\left(s_{i};-\mu_{\text{T}},\sigma_{\text{ext}}\right)ds_{i}} \\ =\log\frac{1}{N}\sum_{\hat{L}=1}^{N} \left[\frac{\int G\left(x_{\hat{L}};s_{\hat{L}},\sigma_{\hat{L}}\right)G\left(s_{\hat{L}};\mu_{\text{T}},\sigma_{\text{ext}}\right)ds_{\hat{L}}\prod_{i\neq\hat{L}} \int G\left(x_{i};s_{i},\sigma_{i}\right)G\left(s_{i};-\mu_{\text{T}},\sigma_{\text{ext}}\right)ds_{i}}{\int G\left(x_{\hat{L}};s_{\hat{L}},\sigma_{\hat{L}}\right)G\left(s_{\hat{L}};-\mu_{\text{T}},\sigma_{\text{ext}}\right)ds_{\hat{L}}\prod_{i\neq\hat{L}} \int G\left(x_{i};s_{i},\sigma_{i}\right)G\left(s_{i};-\mu_{\text{T}},\sigma_{\text{ext}}\right)ds_{i}}\right] \\ =\log\frac{1}{N}\sum_{\hat{L}=1}^{N} \frac{\int G\left(x_{\hat{L}};s_{\hat{L}},\sigma_{\hat{L}}\right)G\left(s_{\hat{L}};\mu_{\text{T}},\sigma_{\text{ext}}\right)ds_{\hat{L}}}{\int G\left(x_{\hat{L}};s_{\hat{L}},\sigma_{\hat{L}}\right)G\left(s_{\hat{L}};-\mu_{\text{T}},\sigma_{\text{ext}}\right)ds_{\hat{L}}} \\ =\log\frac{1}{N}\sum_{\hat{L}=1}^{N} \frac{\int\exp\left[-\frac{\left(x_{\hat{L}}-s_{\hat{L}}\right)^{2}}{2\sigma_{\hat{L}}^{2}}-\frac{\left(s_{\hat{L}}-\mu_{\text{T}}\right)^{2}}{2\sigma_{\text{ext}}^{2}}\right]ds_{\hat{L}}}{\int\exp\left[-\frac{\left(x_{\hat{L}}-s_{\hat{L}}\right)^{2}}{2\sigma_{\hat{L}}^{2}}-\frac{\left(s_{\hat{L}}+\mu_{\text{T}}\right)^{2}}{2\sigma_{\text{ext}}^{2}}\right]ds_{\hat{L}}} \\ =\log\frac{1}{N}\sum_{\hat{L}=1}^{N} \frac{\exp\left[-\frac{\left(x_{\hat{L}}-\mu_{\text{T}}\right)^{2}}{2\left(\sigma_{\text{ext}}^{2}+\sigma_{\hat{L}}^{2}\right)}\right]}{\exp\left[-\frac{\left(x_{\hat{L}}+\mu_{\text{T}}\right)^{2}}{2\left(\sigma_{\text{ext}}^{2}+\sigma_{\hat{L}}^{2}\right)}\right]} \\ =\log\frac{1}{N}\sum_{\hat{L}=1}^{N} \exp\left[\frac{\left(x_{\hat{L}}+\mu_{\text{T}}\right)^{2}-\left(x_{\hat{L}}-\mu_{\text{T}}\right)^{2}}{2\left(\sigma_{\text{ext}}^{2}+\sigma_{\hat{L}}^{2}\right)}\right] \end{matrix}$$

**Constrained fitting of the lapse rate parameter**

If we do not constrain the lapse rate parameter in the Bayesian models, then these models may explain away decision suboptimalities by overestimating lapse rates. To avoid this unwanted flexibility, we use data from the discrimination task to obtain independent estimates of subjects’ lapse rates and then use this information to constrain lapse rates in the models for the visual search task. The logic behind this approach is that lapse rates in the discrimination task can be estimated quite accurately and there is no reason to assume that the frequency of attentional lapses is very different in the visual search task. (One may argue that subjects guess more often when the task is more difficult. However, a difficulty-driven increase in guessing is likely to be due to a higher frequency of non-informative decision variables, rather than due to a higher frequency of attentional lapses. Models should account for this by processes that deteriorate the decision variable, not by increasing the frequency of attentional lapses.) We estimate each subject’s lapse rate by fitting a cumulative Gaussian with a free lapse rate parameter to their discrimination task data. According to these estimates, subjects guessed on 3.2±1.2% of the trials. We fit a beta distribution to the distribution of all individual lapse rate estimates and use this distribution as a prior on the lapse rate in the Bayesian models for the visual search task. The parameters of the beta distribution are *α*=0.18 and *β*=5.61.

**Constrained fitting of sensory noise level parameters**

If we fit sensory noise parameters *σ*_low_ and *σ*_high_ in an entirely unconstrained manner, then we may be giving models an opportunity to explain away decision suboptimalities by overestimating sensory noise levels. We can reduce such flexibility by constraining parameters *σ*_low_ and *σ*_high_ with prior information about the expected sensory noise levels in our experiment. Prior to performing the visual search experiment, each subject performed a discrimination task with an ellipse stimulus identical to the ones used in the visual search task. Due to the simplicity of the discrimination task, our estimates of *σ*_low_ and *σ*_high_ in that task (Table 2 in main text) are probably highly representative of the true noise levels in that task. Unfortunately, however, these estimates may not be representative for the noise levels in the visual search task, because it used a different set size (4 instead of 1) and previous work has shown that sensory noise levels may increase with set size, up to a factor of two [1,2]. We express this increase as ratios, $\frac{\sigma_{\text{low, }N=4}}{\sigma_{\text{low, }N=1}}$ and $\frac{\sigma_{\text{high, }N=4}}{\sigma_{\text{high, }N=1}}$ . To get an estimate of a plausible range for these ratios, we performed a control experiment that includes discrimination tasks at both set sizes 1 and 4.

*Control experiment.* Twelve subjects performed the original discrimination task (as described in the Main text) and a variant of the task with four stimuli. In the variant, subjects were presented on each trial with four stimuli with mixed reliabilities, such that the visual characteristics were identical to the visual search task. One of the stimuli would disappear after 67ms and the task of the subject was to indicate the direction of tilt of the disappeared stimulus. Hence, just as in the visual search experiment, subjects had to encode four stimuli on each trial, because they would not know which of them would disappear. Each subject was tested on two versions with set size 4. In the first version, the stimuli were representative of the visual search task with 0% uncertainty (“lowest heterogeneity”). In the second version, stimuli were representative of the visual search task with 15% uncertainty (“highest heterogeneity”). Each subject performed 300 trials of the original discrimination task and 300 trials of each version of the variant with a set size of 4. The probed stimulus had low reliability in half of the trials and high reliability in the other half.

*Constructing the prior distributions on σ*_low_ *and σ*_high_*.* We fitted cumulative Gaussians to each subject’s data to obtain estimates of *σ*_low_ and *σ*_high_ in all three versions of the discrimination task. Thereafter, we computed for each subject the ratio between the estimated noise levels at set sizes 4 and 1. Across all estimates, we find that the average ratio was 1.36 with a standard deviation of 0.39. A Bayesian ANOVA provided no strong evidence for an effect of reliability (low *vs.* high; BF_inclusion_=0.80) or heterogeneity (lowest *vs*. highest; BF_inclusion_=1.36) on the estimated ratio. Therefore, we will impose a single prior distribution to all sensory noise parameter estimates, regardless of stimulus reliability or level of external uncertainty. We choose to do this by using a Gaussian prior with a mean of 1.36 and a standard deviation of 0.39 on the ratio between the noise levels in the visual search models and the estimated noise levels from the discrimination task.

**REFERENCES**

1. Mazyar H, Van den Berg R, Seilheimer RL, Ma WJ. Independence is elusive : Set size effects on encoding precision in visual search. J Vis. 2013;13: 1–14. doi:10.1167/13.5.8.doi

2. Mazyar H, van den Berg R, Ma WJ. Does precision decrease with set size? J Vis. 2012;12: 10. doi:10.1167/12.6.10
