# Supplementary figures and images for: Imperfect Bayesian inference in visual perception
Source: PLoS Comput Biol. 2019 Apr 18;15(4):e1006465. doi: 10.1371/journal.pcbi.1006465 (PMC6472731; doi:10.1371/journal.pcbi.1006465)

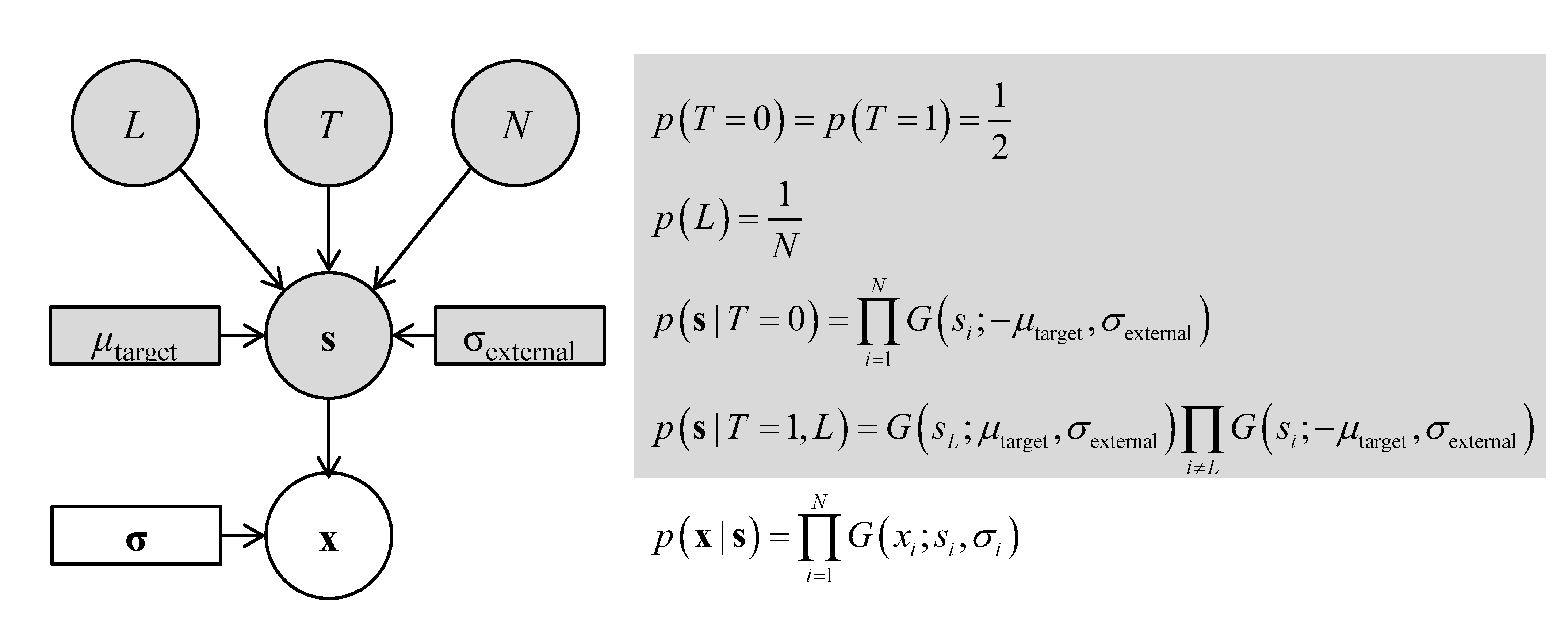

Supplement: S1 Fig — Circles represent random variables, rectangles represent constants, and arrows represent causal relationships. Gray shades represent variables and constants that are under control of the experimenter. On each trial, N = 4 stimuli are presented to the observer. A target is either absent (T = 0) or present (T = 1) among these stimuli. Each location L ϵ {1, 2, …, N} has equal probability of containing the target on target-present trials. On target-absent trials, each stimulus orientation, si, is drawn from the distractor distribution, which is a Gaussian with a mean −μtarget and a standard deviation σexternal. On target-present trials, the stimulus at the target location is drawn from a Gaussian distribution with mean μtarget and standard deviation σexternal, while the remaining N−1 stimuli are drawn from the distractor distribution. We assume that stimulus observations are corrupted by Gaussian noise, such that each observation, xi, is a Gaussian random variable with mean si and standard deviation σi. (TIF) [file pcbi.1006465.s002.tif]

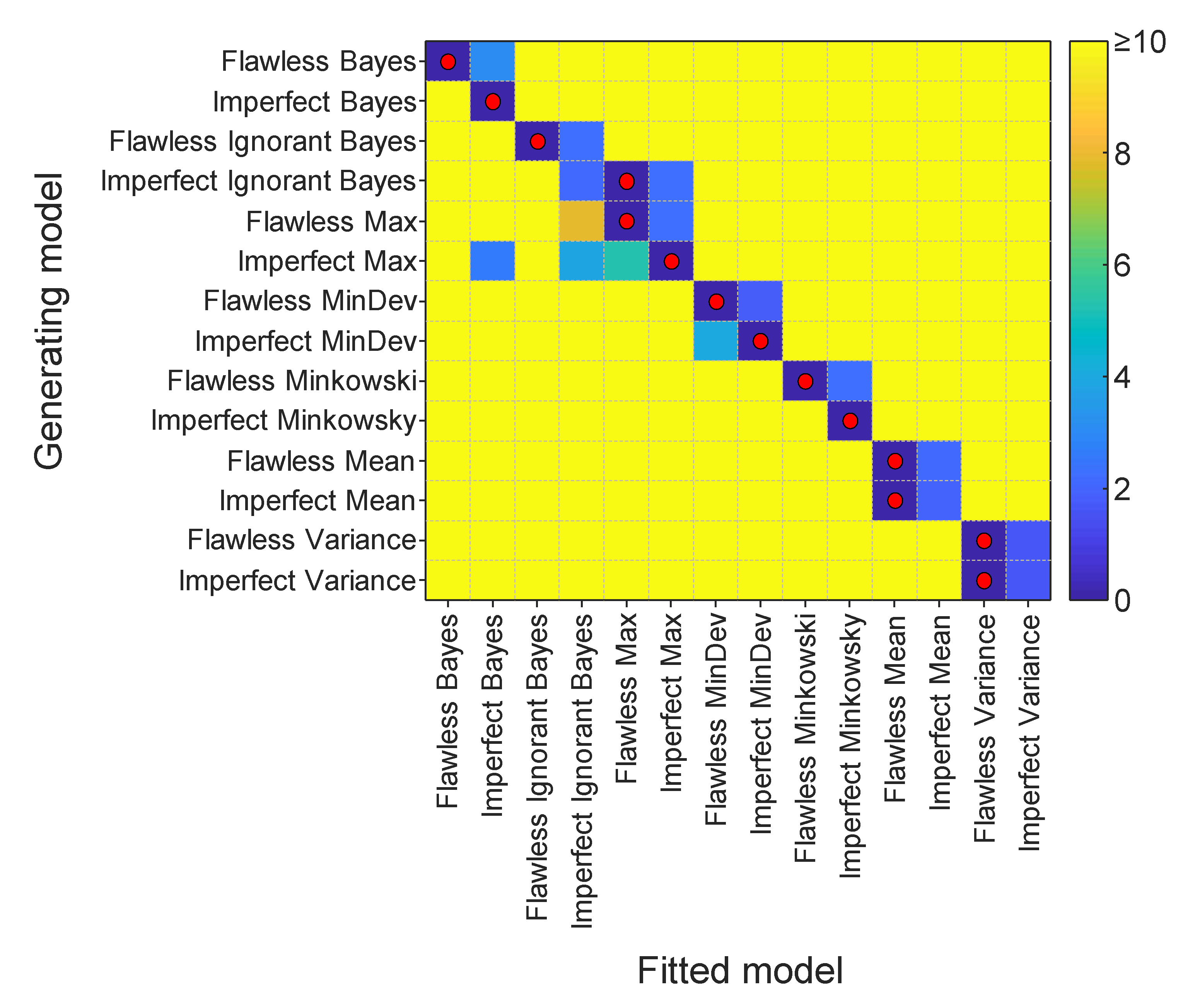

Supplement: S2 Fig — Ten synthetic datasets were generated from each model, by simulating its responses in trials from the condition with 10% external uncertainty. Each dataset had the same number of trials as a subject dataset. Parameter values were drawn from a multivariate Gaussian distribution with the same mean and covariance as the maximum-likelihood estimates obtained from fitting subject data. Hence, the synthetic datasets had the same size and similar statistics as empirical datasets. Each model was fitted to each of the 140 synthetic datasets. The matrix shows for each generating model the average AIC value (across all ten generated datasets from the model) relative to the best-fitting model. In each row, the overall best-fitting model is indicated with a red dot. In most cases, the generating model is the best-fitting model (red dots on diagonal) and most other models are rejected. There are a few wrong classifications (red dots off-diagonal), which indicates that some model pairs cannot reliably be distinguished from each other. Importantly, the model that was most successful in accounting for subject data—the Imperfect Bayesian model—never is selected as the preferred model when data were generated from another model. Hence, it is unlikely that the success of the Imperfect Bayesian model on empirical data was caused by it being overly flexible. (TIF) [file pcbi.1006465.s003.tif]

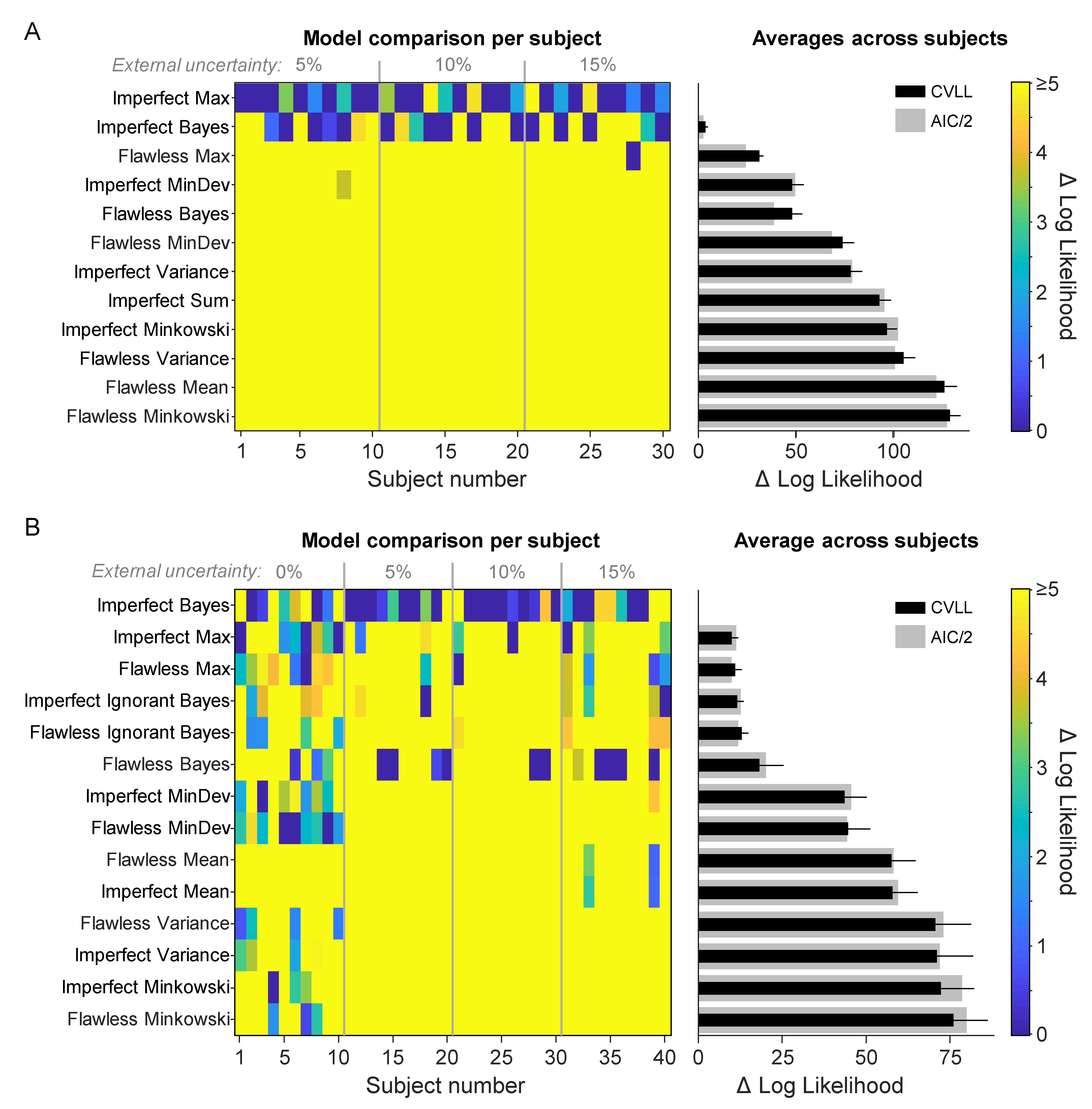

Supplement: S3 Fig — Each individual dataset was fitted 5 times. In each of these fits, a different subset of 20% of the trials was left out. The log likelihood of these left out data were computed using the maximum-likelihood estimates obtained from fitting the other 80% of the data. We summed the 5 log likelihood obtained for each subject to compute a single “cross-validated log likelihood” (CVLL). (A) Results from fitting the conditions with unlimited display time. (B) Results from fitting the conditions with brief display time. (TIF) [file pcbi.1006465.s004.tif]
